# Flexible and Transparent Substrates Based on Gold Nanoparticles and TiO_2_ for in Situ Bioanalysis by Surface-Enhanced Raman Spectroscopy

**DOI:** 10.3390/bios9040145

**Published:** 2019-12-17

**Authors:** Luisa Mandrile, Andrea Mario Giovannozzi, Alessio Sacco, Gianmario Martra, Andrea Mario Rossi

**Affiliations:** 1 Physical Chemistry and Nanotechnologies Group, National Institute of Metrological Research, Strada delle Cacce 91, 10135 Turin, Italy; l.mandrile@inrim.it (L.M.); a.giovannozzi@inrim.it (A.M.G.); a.sacco@inrim.it (A.S.); gianmario.martra@unito.it (G.M.); 2 Department of Chemistry and Interdepartmental Centre NIS, University of Turin, Via Giuria 7, 10125 Turin, Italy

**Keywords:** surface enhanced Raman scattering, flexible substrate, in situ measurements, pesticide, titanium dioxide

## Abstract

Flexible and transparent substrates are emerging as low cost and easy-to-operate support for surface-enhanced Raman spectroscopy (SERS). In particular, in situ SERS detection approach for surface characterization in transmission modality can be efficiently employed for non-invasive analysis of non-planar surfaces. Here we propose a new methodology to fabricate a homogenous, transparent, and flexible SERS membrane by the assistance of a thin TiO_2_ porous layer deposited on the PDMS surface, which supports the uniform loading of gold nanoparticles over large area. The substrate was first characterized for homogeneity, sensitivity and repeatability using a model molecule for SERS, i.e., 7-mercapto-4-methylcoumarin. Satisfactory intra-substrate uniformity and inter-substrates repeatability was achieved, showing an RSD of 10%, and an analytical sensitivity down to 10 nM was determined with an EF of 3.4 × 10^5^ ± 0.4 × 10^5^. Furthermore, SERS detection of pyrimethanil (PMT), a commonly employed pesticide in crops for human consumption, was performed in situ, exploiting the optical transparency of the device, using both model surfaces and non-flat bio-samples. PMT contamination at the phytochemical concentration levels corresponding to commonly used infield doses was successfully detected on the surface of the yellow *Ficus benjiamina* leaves, supporting the use of this substrate for food safety in-field application.

## 1. Introduction

The plasmonic properties of metal nanoparticles can be exploited to produce an intensity enhancement to improve the sensitivity of Raman spectroscopy. Noble metal nanostructures with a localized surface plasmon resonance (LSPR) in the visible spectral range are widely applied in bio-analytical sensing and imaging analysis because of their ability to induce a local intensification of the electromagnetic field, which produces an enhancement in the Raman response of molecules attached or close to their surface [1]. Molecules in close proximity of a plasmonically active surface, as roughened metal nanostructures, experience an enhanced local electromagnetic field and their resulting Raman signals are hugely increased in intensity [2]. Surface-enhanced Raman scattering (SERS) shows as a great potential food analysis, and several studies regarding the identification of pesticide traces in food matrix or on food surface have already been published in literature [3]. The possibility to detect pesticide in fruit samples using different metal nanoparticles (NPs) was recently reported [4,5], in which NPs were deposited on the contaminated surface. In other cases solid surface-based substrates were employed to detect pesticides recovered from homogenized peel samples or using surface swab methods [6]. Some of the most rapid and simple strategies rely on the immobilization of wet-chemically synthesized metal nanoparticles (NPs) on a planar and optically inert substrate such as glass, quartz or silicon [7]. This approach is usually preferred to other high-cost/time-consuming techniques based on e.g., electron-beam lithography and atomic layer deposition [8], even if they suffer sometimes in terms of reproducibility and precision of the location of the nanostructures. Based on their promising optical behavior, the development of facile methods to attach metal nanostructures in a close-pack configuration and precise location on solid substrates represents a florid research field [9]. Recently, several methods for SERS substrates preparation have been proposed [10]. However, beside a great effort devoted to an accurate control of the position and the density of hot spots, increasingly desired properties for SERS devices are flexibility and transparency for daily life applications [11]. Flexible SERS substrates present some desirable features compared to conventional rigid substrates in terms of cost and applicability, because their unique mechanical features enable new functionalities and on-board applications. For instance, this is the case of in situ testing of non-planar contaminated surfaces [12,13], which is a particularly important feature in some fields, such as art works and food analysis. Furthermore, the flexible plasmonics research field has provided good advancements in the recent years on the design and fabrication of diverse sustainable chips for daily life applications [14,15,16]. The efficacy and costs of plasmonic sensors depend on raw materials, fabrication process time, and critical control points.

Polydimethylsiloxane (PDMS) is a polymer material that provides interesting features for SERS substrates thanks to its high transparency, elasticity, chemical stability, and adhesion properties [17,18]. Several fabrication protocols have been reported in literature so far for flexible substrates, both based on bottom-up self-assembly [19,20] or top down lithography [21,22,23]. In view of practical SERS application in analytical and bioanalytical chemistry, it is worth to remark that the intra-substrate uniformity and the inter-substrates repeatability of the signal intensity must be optimized, aiming at a good compromise with SERS efficiency [21]. In accordance with the recent literature, relative standard deviation (RSDs) of the SERS signal intensity close to 20% is considered a good result [24,25]. However, substrates with RSD values around 10% are would be desirable for quantification purposes [26]. Although these SERS substrates guarantee high sensitivity in the detection of chemical species of interest, they sometimes lack of reproducibility and high variability when point-to-point comparisons are performed, preventing accurate and precise quantification. Therefore, the best compromise between sensitivity and repeatability of SERS strategies have to be found to obtain reliable analytical methods for real applications.

In the current work, a low cost, simple, fast, and reproducible fabrication method for SERS substrates is proposed based on spheroidal AuNPs dispersed on a layer of TiO_2_ nanoparticles coating a PDMS film. The most advantages of the proposed substrate lays in the facility of preparation and the low cost of the procedure compared to flexible plasmonic substrates already proposed in literature, without the need of any particular technology. The flexible and semi-transparent substrates are tested in terms of morphology, homogeneity, repeatability of analytical results and SERS efficiency. 7-mercapto-4-methylcumarin (MMC) is used as Raman reporter for the characterization tests and SERS maps are collected to determine surface uniformity. Furthermore, an application study was performed, detecting traces of a pesticide (namely pyrimethanil ((PMT), N-(4,6-dimethylpyrimidin-2-yl)-phenylamine)) [27], on model surfaces and real biological surfaces, such as fresh leaves. Thus it was proved that the preparation procedure here designed and optimized allowed to obtain an easy-handling and efficient SERS device with the typical flexibility and adhesiveness of PDMS.

## 2. Materials and Methods

### 2.1. Chemicals and Materials

Chemicals and materials employed for the preparation and testing of the SERS supports were the following: Hydrogen tetrachloroaurate trihydrate (HAuCl_4_ 3H_2_O ≥ 99%), trisodium citrate dihydrate (≥99%), from Sigma-Aldrich (Milan, Italy); sodium hydroxyde (NaOH 97%), hydrochloric acid (HCl 37%), nitric acid (HNO_3_ 68%), absolute ethanol (99.99%), acetone (99.99%), and hydroxylamine hydrochloride (H_3_NO⋅HCl, 99%) from Novachimica (Milano, Italy); Scala^®^ (400 g/L of pyrimethanil suspension) from BASF Italia (Volpiano, Italy); Sylgard 184 elastomer with its curing agent (*w*/*w* = 10:1) kit for PDMS preparation was purchased from Sigma Aldrich; AEROXIDE^®^ TiO_2_ P25 nanopowder was purchased by Evonik (declared NPs size 20 ± 5 nm [28]); 7-mercapto-4-methylcumarin 99% of purity from Sigma-Aldrich. All solutions were prepared with Milli-Q quality water (18 MΩcm); silicon wafers with a 300 nm of silicon dioxide layer on top were purchased from Si-Mat (Kaufering, Germany). The *Ficus benjiamina* leaves used for the assays were taken from plants cultivated at the National Institute of Metrological Research (Turin, Italy).

### 2.2. Gold Nanoparticles Preparation

All glassware used in the experiment was soaked in aqua regia (HCl:HNO_3_ 3:1 *v*/*v*), rinsed in water and dried with nitrogen before use. Spheroidal AuNPs with a nominal diameter of 40 nm were synthesized according to Frens G, 1973 [29]. Briefly 5 mL of a 1% aqueous solution of trisodium citrate were rapidly injected into 500 mL boiling solution of HAuCl_4_ (0.01% *v*/*v*) for the preparation of 40 nm AuNPs. The mixture was further refluxed for 10 min and then cooled to room temperature under continuous stirring. Larger AuNPs with a diameter of 120 nm were obtained via seed-mediated growth 40 nm AuNPs, using an optimized growing procedure based on hydroxylamine hydrochloride. In detail, 4 mL of Au seeds suspension were put into a round-bottom flask with 53.8 mL of Milli-Q water under continuous stirring and the different solutions were added in the following order: 920 µL of 1% *v*/*v* aqueous solution of trisodium citrate (stirring for 3 min), 1.4 mL of 10 mM hydroxylamine hydrochloride solution (stirring for 8 min) and 90 µL of 10% *w*/*v* HAuCl_4_ (added dropwise, 1 drop per second). The suspensions were kept in continuous stirring overnight at room temperature in the dark before using it. See Supporting Information (Figures S1–S3) for major details about the characterization of the synthetized AuNPs.

### 2.3. Preparation of Pesticides Standard Suspensions and Solutions

Pyrimethanil stock standard suspension was prepared by diluting commercial Scala^®^ (400 g L^−1^ of pyrimethanil suspension) in Milli-Q water, to reach a concentration of 8 g L^−1^ and 4 g mg L^−1^. Pyrimethanil standard solutions were prepared by subsequent dilutions from the stock suspensions in water to reach the following concentrations: 40 mg L^−1^, 30 mg L^−1^, 20 mg L^−1^, 10 mg L^−1^, 5 mg L^−1^, and 1 mg L^−1^. These pure pyrimethanil standards were used in SERS test on model surface and on real biological samples.

### 2.4. Flexible SERS Substrate Preparation

**Figure 1:**
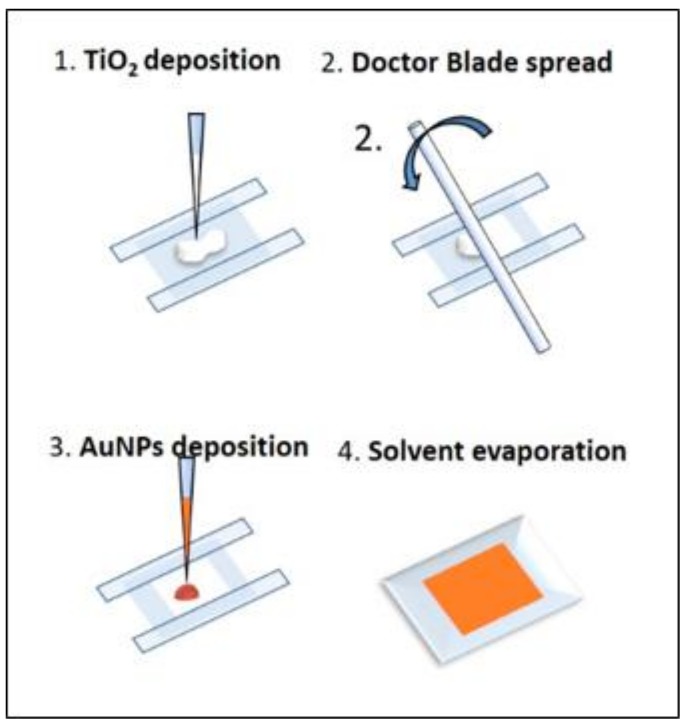
Polydimethylsiloxane (PDMS)-based surface-enhanced Raman spectroscopy (SERS) substrate graphic preparation scheme.

The fabrication procedure is schematically depicted in Figure 1. First, the PDMS matrix was prepared by mixing the Sylgard 184 elastomer with its curing agent (*w*/*w* = 10:1). Afterwards the polymeric mixture was degassed in vacuum for 40 min and poured on a clean glass petri-dish to obtain the desired thickness and cured in a microwave oven. The Design of Experiments (DoE) approach was used to determine the best conditions to obtain the desired features of the film. Three factors are considered in the optimization of the PDMS substrate preparation, i.e., the PDMS thickness (0.2 mm–2 mm), the curing time (3 min–15 min) and the microwave power (300 W–700 W). The experimental details with the corresponding results is reported in Supplementary Information. Final preparation procedure was defined for 1 mm PDMS film cured for 15 min at 700 W and cooled down to room temperature. TiO_2_ paste is prepared by mixing Aeroxide^®^ P25 titanium dioxide powder in ethanol (EtOH). The suspensions were then homogenized for 10 min in ultra-sonic bath at 15 °C and at a power of 80 W to obtain stable and well de-agglomerated suspensions. The titanium dioxide paste was then used to prepare a thin porous film on the PDMS substrate by manual doctor blade deposition with the help of a glass stick used as a roller-pin [30]. A similar optimization approach was used to determine the best TiO_2_ deposition conditions. In this case the evaluated factors were TiO_2_ paste concentration (5–20%) and dropped volume (5 µL–15 µL). The operative conditions were kept constant in accordance with doctor blade published methodology [31]. Also in this case arbitrary appreciation scales are defined and performance indexes are attributed to different experimental results to evaluate the responses. Then a MRL model was calculated. The monitored responses were flexibility, i.e., absence of fractures on the dried films, adhesion, and water diffusion across the layer, responsible for AuNPs homogenous distribution. The list of experiments is reported in Table S3 with the associated responses, replicates are included to test reproducibility and strengthen the model. A mask defining an area of 0.5 × 0.5 cm^2^ is placed on PDMS film and 10 µL of TiO_2_ are dropped on the PDMS, the glass stick is rolled with a rapid movement on the surface applying a light pressure to spread the paste and remove the excess. The concentration and volume of the paste are optimized using DoE approach and MLR model. Optimized procedure consists in spreading 8 µL of 10% *w*/*w* TiO_2_ paste. AuNPs with nominal diameter of 120 nm are synthetized accordingly with procedure described in Section 2.2. After the synthesis they are concentrated and re-dispersed in EtOH to a final concentration factor of 1:20, by centrifugation (10 min, 3000 rpm) and removal of the clear supernatant. 10 µL of concentrated AuNPs\EtOH are dropped on the TiO_2_ square pad. The 120 nm AuNPs diffuse through the pores and micro-channels of the titanium dioxide layer and EtOH evaporates very rapidly provoking a uniform covering of the surface. After drying, the substrates are conserved in dark conditions.

### 2.5. SEM Imaging

SEM images of the flexible SERS substrate were collected using Inspect F (FEI, Hillsboro, Oregon), operated at 10 kV. Samples were covered with 10 nm of Au/Pd alloy using sputtering technology to overcome problems due to the high electrical insulating property of PDMS polymer. Back scattered electrons detector was used to maximize contrast between Au and TiO_2_ nanoparticles.

### 2.6. SERS Mapping Measurements

SERS spectra were recorded using a Thermo Scientific DXRXi Raman Imaging equipped with a 20× long working distance objective, a 780 nm excitation laser source, an automatic sample stage, and a charge-coupled device (CCD) detector. Raman equipment is monthly calibrated through a software-controlled calibration tool containing a neon tube for wavelength calibration, a polystyrene standard for frequency calibration check and a white light sources for intensity calibration. A frequency uncertainty of 5 cm^–1^ is determined by the grating resolution (grating groove density is 1200 lines/mm), and an intensity uncertainty lower than 5% is associated to the instrument. Laser power of 2 mW is used and a spectral range from 50 cm^−1^ to 3400 cm^−1^ is investigated. The acquisition time for each spectrum was 0.001 s (100 Hz acquisition frequency) for 20 exposures. Raman maps of 0.25 mm^2^ are collected, image pixel size is 25 µm, spot size 1.9 µm with a 20× long working distance objective. Different Raman mapping experiments were designed to attest the suitability of the obtained flexible SERS substrate, all the experimental conditions of the mentioned experiments are described and discussed in the following section.

### 2.7. SERS Characterization Tests

The optimized flexible SERS substrates were tested by using 7-mercapto-4-methylcoumarin (MMC) in ethanol as a Raman reporter molecule. To this aim, the SERS substrates were incubated in 1 mM MMC in ethanol solution for 4 h and then abundantly rinsed with ethanol to remove the MMC in excess on the surface. The incubated substrates were then mapped to collect SERS signals of the reporter molecule after its binding with the surface of the AuNPs spread on the flexible substrate.

### 2.8. Enhancement Factor Calculation

The Raman enhancement factor (EF) was calculated following literature methods [32] from the formula in Equation (1):

EF = (I_SERS_/N_SERS_)/(I_Raman_/N_Raman_) (1)

where I_SERS_ is the SERS intensity of the characteristic peak of MMC at 1595 cm^−1^ registered on the SERS substrate, N_SERS_ is the number of molecules responsible of I_SERS,_ I_Raman_ is the intensity of the characteristic peak of MMC at 1595 cm^−1^ registered in a 0.01 M solution and N_Raman_ is the number of molecules contained in the confocal volume of the excitation laser during the Raman measurement and contributing to I_Raman_. To determine the value of I_Raman_ a 0.01 M ethanol solution of MMC was poured into a microwell and analyzed with a 20 × LWD objective, using an excitation wavelength of 780 nm with a power of 8 mW (20 s exposure time), 5.2 counts/s (I_Raman_) were measured for the 1595 cm^–1^ peak. Using the measured interaction volume of 68 μm^3^, the number of molecules responsible for the Raman signal (N_Raman_) was estimated to be 4.1 × 10^9^. To determine the value of I_SERS_ a SERS substrate coated with AuNPs was incubated in 4 mL of MMC 10^−4^ M for 4 h, then abundantly rinsed and dried. Three Raman maps were collected on 6 different samples using identical conditions as for I_Raman_ and a signal intensity of (2200 ± 400) counts/s was measured for the 1595 cm^−1^ peak. The number of molecules inside the laser spot was estimated by assuming a monolayer of MMC over the AuNPs surface. Knowing the AuNPs concentration in EtOH suspension (2.7 × 10^−10^ mol/L) and the average diameter of one spheroidal NP (116 ± 11 nm), obtained from the analysis of 100 NPs diameters by SEM, and hypothesizing a uniform distribution of AuNPs on the surface (as proved by SEM imaging and SERS homogeneity tests) and a laser spot size with diameter 1.9 μm, an effective active area available for SERS (2.7 ± 0.5 µm^2^) was calculated. This gives an estimate of N_SERS_ = 4.2 × 10^6^ ± 0.3 × 10^6^ molecules.

## 3. Results and Discussion

### 3.1. Flexible SERS Substrate Optimization

The preparation protocol of the plasmonic flexible substrate was defined after several characterization tests aimed at defining its performances. The PDMS curing procedure and the thickness of the TiO_2_ films were optimized by DoE approach to find the best compromise between mechanical properties, adhesiveness and flexibility (see Figures S3–S5 in Supplementary Information). The longer and more energetic was the curing, the more the PDMS film was resistant. Conversely, very sticky PDMS was obtained with shorter and less energetic curing, as it was expected. For this specific application, i.e., the fabrication of a flexible SERS substrate to be laid down on testing surfaces, the PDMS should by sticky enough to adhere on the testing surface, but sufficiently consistent to allow easy handling with tweezers. The final protocol for an optimal PDMS substrate preparation consists in drop casting an amount of liquid PDMS in a petri dish adequate to obtain 1 mm thick PDMS layer, whose curing was carried out at maximum power (700 W) for 15 min. In this way reproducibly sticky but consistent substrates were obtained. An optimization study was also conducted for the TiO_2_ layer deposition (see Figures S7 and S8). As the basis of the experimental results in SI show, 8 µL of a TiO_2_ suspension in 10% EtOH were selected as optimized preparation conditions for the deposition of a titanium dioxide film on the PDMS film.

The TiO_2_ layer behaves as a diffusion agent supporting the uniform distribution of the AuNPs on the SERS substrate, because of the presence of micro channels and pores. The 3D matrix, created into the thin titanium dioxide layer after the evaporation of the solvent, drives the AuNPs over the covered area and provides a uniform distribution of the NPs on the surface. This effect was empirically observed. In absence of the titanium dioxide porous layer, the AuNPs drop evaporates leaving the classical coffee stain shape on the surface (Figure S9). This condition is highly undesired since it represents a non-homogeneous distribution of AuNPs over the surface, impairing the uniformity of response of the device. The homogeneous AuNPs distribution over the surface is a function of the TiO_2_ layer thickness, which depends on the amount of TiO_2_ deposited on the surface: The thicker the TiO_2_ layer, the better the AuNPs colloid is distributed on the surface. However, if a very thick titanium dioxide layer is deposited on the PDMS, transparency and sticky properties are lost (Figure S10). Moreover, compared to a thin layer, a thick film provokes the formation of fractures and wrinkles on the surface during manipulation. Optimization of such parameter was then fundamental to reach high optical homogeneity, while preserving flexibility, transparency and useful adhesion properties on model test substrates. The final thickness of TiO_2_ layer after drying ranges from 0.7 to 1.3 µm.

The density of AuNPs on the substrate, was calculated on the basis of AuNPs concentration (1,4 10–10 mol/L) and the approximated total volume of the TiO_2_ layer (1 × 10^8^ µm^3^) providing a density of 8,4 NPs/µm^3^. From the SEM image in cross section (Figure S11), we know that the distribution is not uniform in the TiO2 film thickness, but the NPs are mostly concentrated on the surface. Some AuNPs agglomeration cannot be excluded after concentration and drying process, however this conditions is suitable to increase the “hotspot” concentration that are formed in the region between two or more NPs, contributing to the Raman enhancement. The Au NPs used in this study were stabilized by citrates, forming adducts exposing one or two carboxylic/carboxylate groups towards the exterior. These groups can act as stronger ligand than water molecules usually coordinated to the Ti^4+^ sites exposed at the surface of the (101)-type surface terminations, overwhelmingly terminating the TiO_2_ nanoparticles used for the fabrication of the substrate. Even though, in practical use the substrate is intended to be used once, evidence of the stability of the substrate was obtained by its resistance to soaking, rinsing and application on various surfaces.

### 3.2. SERS Substrate Characterization

**Figure 2:**
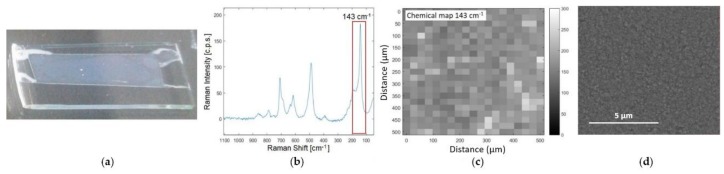
Characterization of the titanium dioxide porous layer deposited on PDMS after drying. (**a**) Visual appearance of the TiO_2_ film on PDMS; (**b**) mean spectrum of the entire map calculated by averaging the spectra corresponding to each pixel of the map; (**c**) Raman chemical map, in which the degree of grey is associated to the intensity of 143 cm^–1^ peak; (**d**) the SEM image of the TiO_2_ film on PDMS.

The optimized substrates were characterized to determine the homogeneity of the active surface. For the characterization tests the analytical configuration frontal analysis was adopted, i.e., the active side of the substrate faced towards the objective, and the Raman excitation laser was focalized directly on the coated side. First, the uniformity of the TiO_2_ layer was tested by Raman mapping as shown in Figure 2. The intensity of the most characteristic peak of TiO_2_ anatase phase, at 143 cm^−1^ due to the Eg mode [33], shows a RSD < 15% over an area of 500 × 500 µm^2^, providing proof of a satisfactory homogeneity of the porous film on the PDMS surface. Moreover, the absence of cracks and defects at the micrometric level was evaluated by SEM images (Figure 2c), which demonstrate a good uniformity of the TiO_2_ layer.

**Figure 3:**
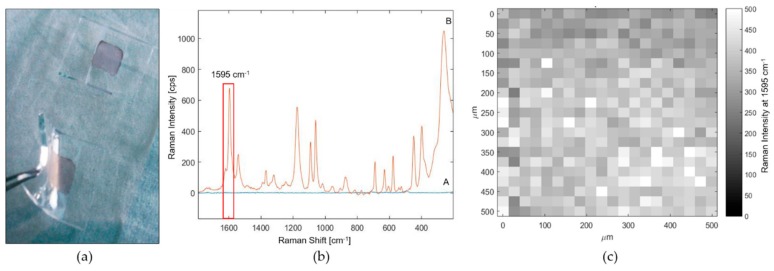
SERS substrate characterization test after incubation in 7-mercapto-4-methylcumarin (MMC) solution. (**a**) optical image of the prepared flexible SERS substrates. (**b**) mean spectra of SERS maps collected on PDMS/TiO_2_ substrate (A) without AuNPs (blue spectrum) and (B) with AuNPs (orange spectrum). (**c**) Raman map associated to the intensity of 1595 cm^−1^ peak of MMC.

Subsequently the distribution of AuNPs was evaluated by direct SEM imaging investigation (SEM images in zenith view and cross section are shown in Figure S11) and by Raman spectroscopy, using MMC as a Raman reporter [34]. This molecule was selected because of its large Raman cross section and its strong chemical affinity for gold due to the sulfhydryl functional group (R-SH). The Raman spectrum of MMC in solid state is shown in Figure S12 with the corresponding peaks assignments. SERS substrates with and without AuNPs were soaked in a 1 × 10^−3^ mM MMC ethanol solution, then abundantly rinsed with EtOH and dried with a gentle nitrogen flux before Raman analysis. In the case of TiO_2_/PDMS substrates, no Raman signals of the MMC molecules were detected (Figure 3b,A), confirming that TiO_2_ NPs do not contribute to SERS enhancement. Conversely, when the TiO_2_/PDMS substrates were loaded with AuNPs, the specific signals of MMC were revealed (Figure 3b,B), indicating the occurrence of a SERS regime, due to the interaction of MMC molecules with AuNPs surface. Thus, monitoring the intensity of the MMC peak at 1595 cm^−1^ by Raman mapping (Figure 3c), a good homogeneity of the AuNPs distribution all over the composite substrate was revealed, as can be also appreciated by the uniform color of the substrates shown in Figure 3a. In more detail, the intra- and inter-maps RSD of the SERS intensity of the 1595 cm^−1^ peak for MMC over an area of 500 × 500 µm^2^ were <25% and <15% respectively, demonstrate that the TiO_2_-assisted distribution of AuNPs is a good method to uniformly spread nanoparticles on a PDMS substrate.

**Figure 4:**
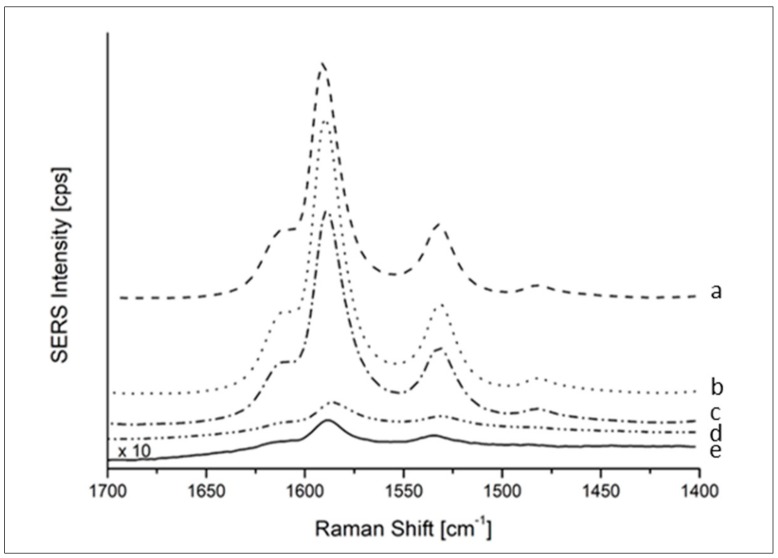
SERS sensitivity test after incubation in progressively more diluted solution of MMC. Mean spectra of a SERS map of 500 × 500 µm^2^ collected on the center of the SERS substrate are shown for each concentration level (**a**) 100 µM, (**b**) 10 µM, (**c**) 1 µM, (**d**) 0.1 µM, and (**e**) 0.01 µM.

In order to test the sensitivity of response of the SERS substrate, this measurement was repeated at progressively lower concentration of MMC, providing a signal higher than the LOD (defined as 3 × S/N) down to 10^−8^ M, as shown in Figure 4. The SERS enhancement factor (EF) was also calculated to provide a quality parameter to compare with other literature SERS systems. The EF is based on the ratio of the SERS signal intensity over the intensity of normal Raman signals, both normalized by the number of molecules responsible of those signals. The calculated EF was to 3.4 × 10^5^ ± 0.4 × 10^5^ which is remarkable in comparison with recent results obtained for much more costly SERS substrates [35].

### 3.3. Homogeneity of Response in Transmission Configuration

**Figure 5:**
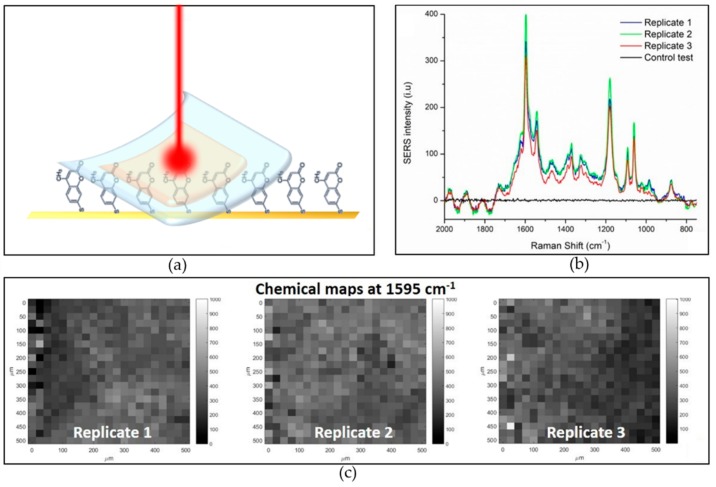
SERS test in use configuration. (**a**) graphical representation of the analytical configuration in transmission; (**b**) mean spectra of three analogue measurements and control test spectrum (black) collected on the contaminated model surface before SERS substrate applications; (**c**) SERS maps, based on the most intense MMC Raman peak, of three replicates, attesting good repeatability and SERS response homogeneity.

The optimized SERS substrate was tested in a real use configuration, i.e., stuck on a contaminated surface (the configuration is represented in the scheme in Figure 5a). The Raman measurement was performed in a transmission configuration with the excitation wavelength passing through the PDMS film and investigating the contaminated surface underneath the SERS substrate. The goal is to confirm the possibility to enhance Raman signals of molecules deposited on a surface after sticking the SERS substrate on it. For this scope a model surface is used, namely a Si chip coated with flat gold incubated in MMC 10^−3^ M for 4 h. Such incubation promotes the formation of a continuous monolayer of MMC molecules on the golden flat surface thanks to the interaction of the thiol group with Au atoms [36]. This contaminated flat model surface does not yield SERS effects because of its low roughness and the absence of LSPR (a control test spectrum collected on the MMC monolayer on the flat gold without the SERS substrate is showed in black in Figure 5b). The SERS substrate perfectly adheres to the flat model surface. The excitation laser is then focalized on the plane of contact between the flat model substrate (covered with one monolayer of MMC molecules) and the active side of SERS substrate, which exposes the AuNPs on the surface. The test was repeated three times to evaluate the reproducibility of the system.

Detailed study about the homogeneity of the SERS response in reverse configuration across the surface was also conducted. The uniformity of the SERS response over an active substrate is a crucial aspect in view of a real application; in order to evaluate the variability of the signal across the substrate, the relative standard deviation (RSD) was considered. For the purpose of defining the SERS substrates uniformity, for analytical and bioanalytical applications over a large area the analysis should be extended to greater portions of the substrate, and the analyzed area should be stated unequivocally. Low RSD values show remarkable homogeneity [37] and very low values have been recently reported in literature [38]. However, a standard protocol to evaluate spatial homogeneity of SERS substrates is still missing. Sometimes the RSD is calculated by considering the average of tens to thousands punctual spectra [39,40], whereas sometimes it is obtained from multiple scanning areas on the substrate [41]. Moreover, the considered area onto which the analysis is carried out is not always declared, leading to not comparable literature results [42,43]. In this study, the intra-map homogeneity, which is described by the RSD calculated on all the spectra composing the Raman map, was first calculated. This parameter represents the variability from pixel to pixel within a single map. As long as the scanned area is enlarged, the response variability increases, leading to higher intra-map RSD values. On the other hand, the repeatability of the measurements on different substrates regions or different substrates (inter-map RSD) can be improved by increasing the area of analysis, in this way local differences are averaged. In punctual confocal Raman, the spot diameter is a constant and it depends on the section of the focalized laser on the investigated surface, which is 2.7 μm for a 780 nm laser and 0.25 NA, 10× objective (operating conditions of this study). However, a spot-size enlargement can be practically obtained collecting a map of spectra on a wider area (500 × 500 µm^2^ in this study) and calculating their mean. In this way, the point-to-point intensity differences can be overcome. The resulting average spectrum is representative for the whole mapped area of 500 × 500 µm^2^. The intra and inter maps RSDs of three replicates are reported in Table 1, confirming that higher repeatability is obtained by mapping a large area, then considering punctual spectra.

In this concern, a relevant information is to define the minimum area that guarantees adequate repeatability. The RSD of repeated measurements of equal areas on the same SERS substrate, i.e., the inter-maps RSD, is considered to evaluate this. A similar approach was previously used by Fu et al. [44]. For this scope, the calculation of the RSDs was performed by considering the SERS spectra acquired over three analogue samples. Three model surfaces coated by a MMC monolayer are covered with a SERS substrate and measured by SERS mapping. For each sample the SERS map is repeated considering progressively increasing areas as reported in Table S4 and Figure S13. As long as the scanned area increases, the variation of the results obtained for three repeated measurements gets lower and lower. An area of 0.25 mm^2^ is demonstrated to be optimal to guarantee good measurement repeatability, since an RSD of 10% is obtained.

### 3.4. Simulation of Application of the SERS Substrate for Pesticides Detection

**Figure 6:**
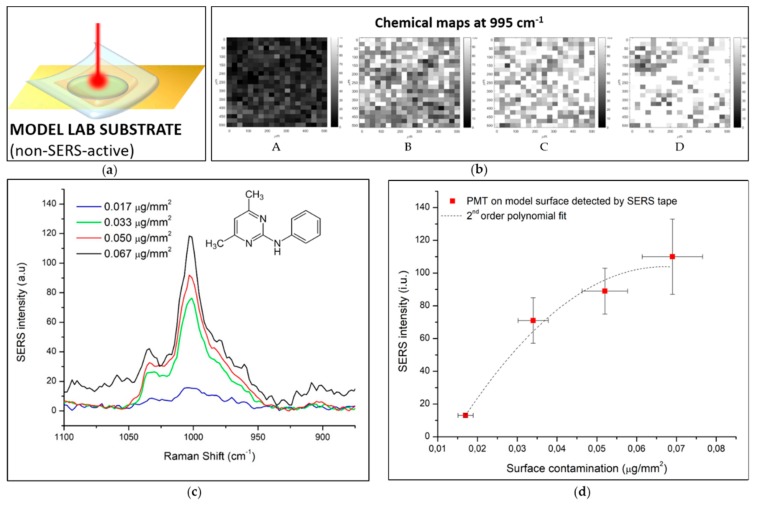
SERS detection of PMT spread on flat model surface by SERS. (**a**) graphical scheme of measurement configuration; (**b**) SERS chemical maps associated to the intensity of 996 cm^−1^ of model surface contaminated at (A) 0.017 µg/mm^2^, (B) 0.033 µg/mm^2^, (C) 0.050 µg/mm^2^, and (D) 0.067 µg/mm^2^; (**c**) average spectra of four repeated measurements for the four examined concentration levels; (**d**) SERS intensity versus contamination level plot with the associated error bars.

**Figure 7:**
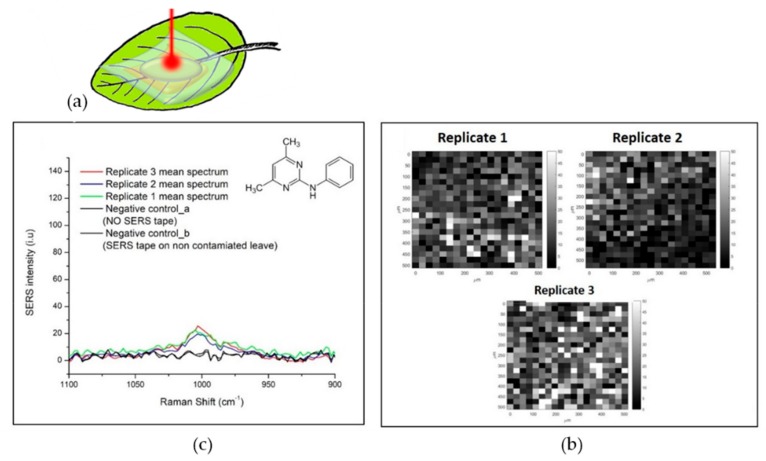
SERS detection of PMT traces on a Ficus Benjamina leaf spiked by drop casting a micro drop of fungicide on the leave page. (**a**) Graphic representation of the experiment configuration; (**b**) Mean spectra of three SERS maps and negative control spectra; (**c**) SERS maps correlated to 996 cm^−1^ peak intensity.

The produced SERS substrate was also tested for a real application in food safety field. In particular pyrimethanil (PMT), a fungicide, was selected as a test material and spread on a model surface (experimental configuration shown in Figure 6a) and on fresh leaves (experimental configuration shown in Figure 7a). In Figure 6, the analytical results of pesticides detection over a flat model surface using the SERS substrate are shown. The flat Au/Si model surface was used for the preliminary tests using the fungicide test molecule in order to better control the surface contamination level. A known amount of PMT was deposited by drop coating a known concentration of PMT suspension on the model surface. After drying in a vacuum chamber, a quite uniform distribution of the contaminant on a well-defined area was obtained, providing a reliable information about the contamination level expressed as µg/mm^2^. The PMT characteristic signal at 997 cm^−1^, that corresponds to the breathing mode of the aromatic ring of the molecule [45], is associated to the grey scale of the chemical maps reported in Figure 6b. The SERS substrate was applied on four increasingly contaminated surfaces and SERS maps were collected. The average intensity of PMT signals in the chemical maps provided an interesting trend of the signal intensity correlated with the contamination level of the investigated area. Each analysis was repeated four times, to verify measurement repeatability. The standard deviation of the four repeated measurements is reported as vertical error bars in Figure 6d. The *x* axis error bars are associated to the B-type contributions to the standard uncertainty associated to the surface contamination. All the terms contributing to the uncertainty associated to the surface contamination level, i.e., the concentration of the starting suspension, the dropped volume and the covered area, are propagated using the Monte Carlo method [46].

## 4. Conclusions

A further step towards real applications was then performed applying the substrate on a real biological sample. Yellow *Ficus benjiamina* leaves were used in this scope as representative test materials. The yellow leaves were spiked with 10 µL of PMT 400 mg/kg (which correspond to the typical concentration used for in field treatments), and analyzed by SERS mapping after the application of the SERS substrate, as shown in Figure 7. Typical Raman signals of the fungicide were detected when the SERS substrate was stuck onto the leaf surface and a light pressure was applied to promote adhesion. A preliminary negative control spectrum, i.e., the Raman spectrum on the contaminated leaf without the SERS substrate, confirmed that the contaminant concentration is too low to be detected with normal Raman, and that a SERS strategy is needed. Secondly, a SERS spectrum of a non-contaminated leaf using the substrate was recorded to exclude interference or false assignments due to the enhancement of other molecular species contained in the biological sample. As it can be noticed, the two control spectra do not show any peak in the spectral region where PMT presents its characteristic peak. After the application of the SERS substrate on the contaminated leaf, the PMT characteristic peak at 997 cm^–1^ is detected, and also a good repeatability in three repeated measurements was obtained, as shown in Figure 7.

In conclusion, AuNPs deposition assisted by a TiO_2_ layer on a polymeric support represents an effective strategy to produce homogeneous, reproducible and active flexible SERS substrates. The mapping of an area of 500 × 500 µm^2^ is sufficient to guarantee measurement repeatability <15%, considering both inter and intra substrate analysis. The time required for each SERS map is about 3 min, which is an adequate measurement time in routine control analysis. The developed device and methodology is ready to be transferred to specific real applications. The revealed trend of SERS intensity as a function of concentration represents the a very promising first step for the development of a quantitative method based on the proposed flexible and transparent device, that will be further investigated in the near future. Further efforts are needed to reduce the uncertainty in order to boost the sensitivity of the method and its resolution before calibration curves can be assessed for quantitative measurements. The SERS substrate has the potential for the detection of a wide range of molecular species of food-safety interest, such as pesticides, non-intentionally added materials, and food packaging contaminants. In particular, a not particularly SERS active molecule was employed in this study, and no specific chemical interaction between the analyte and the AuNPs is present, as we previously demonstrated [4]. This represents an important condition while testing new SERS substrates when a generalization to several analytes and application is foreseen. Moreover the presence of TiO_2_ photoactive nanoparticles as one of the substrate constituents suggests the possibility to photodegrade organic residues on the substrate after analysis for potential multiple reuse. Further studies about the reuse of the substrate after a UV cleaning will be an interesting area of future investigations.

## Figures and Tables

**Table 1:** Inter-maps and intra-map percent relative standard deviation on 1595 cm^−1^ peak of three replicates of MMC monolayer analysis using the SERS substrate.

Intra-Map		
**Replicate**	**Mean (I _1595 cm_^−1^)**	**RSD**
1	311	33%
2	398	21%
3	340	27%
**Inter-map**		
	**Mean (I _1595 cm_^−1^)**	**RSD**
1, 2, 3	350	10.3%

## Supplementary Materials

The following are available online at https://www.mdpi.com/2079-6374/9/4/145/s1, Figure S1: SEM images at magnification 80000× of AuNPs with nominal diameter of (a) 40 nm and (b) 120 nm deposited on a clean silicon wafer, Figure S2: Size distribution of synthetized AuNPs with nominal diameter of (a) 40 nm and (b) 120 nm, calculated by the Gaussian fit of the diameters of 53 and 64 NPs observed by SEM, respectively, Figure S3: Visible absorption spectra of the synthetized AuNPs colloids in water, Figure S4: Optimization of PDMS thickness for the application by DOE: Observed versus predicted (a) mechanical properties and (b) adhesion performance marks calculated by MLR model, Figure S5: MLR model coefficients for (a) mechanical properties of PDMS and (b) adhesion, Figure S6: (a–d) response curves for adhesion and mechanical properties as function of PDMS thickness, curing power and curing time; (e) sweet spot plot for the identification of the best thickness and curing conditions of PDMS tape for SERS substrates application, Figure S7: Observed vs predicted Performance marks for the three monitored responses (a) flexibility; (b) adhesion; (c) diffusion, Figure S8: MLR model coefficients for a) flexibility of TiO2 layer; (b) adhesion of the tape after the deposition of the TiO2 layer; (c) diffusion of AuNPs over the tape, Figure S9: visual appearance of TiO2 covered PDMS tape corresponding to undesired and desired experimental conditions. (a,c) thin and flexible TiO2 layer obtained by depositing 8 µl 10% w/w paste before and after AuNPs deposition respectively; (b,d) thicker TiO2 layer obtained by depositing 15 µl 20% w/w paste, before and after AuNPs deposition respectively, Figure S10: Response curves associate to the three monitored responses, (a) flexibility, (b) adhesion, (c) diffusion; (d) sweet spot plot for the optimization of the titanium dioxide layer, Figure S11: SEM images (back scattered electrons detector, 20 000 × magnification). AuNPs deposited on TiO2/PDMS tape. (a) zenithal view; (b) cross sectional view, Figure S12: Raman spectrum of MMC in solid state with the characteristic peaks assignments, Figure S13: trend of the RSD % calculated inter 3 measurements as a function of the size of the mapped area, Table S1: measured size of AuNPs by SEM, Table S2: table of experiments for PDMS optimization and collection of empiric responses, Table S3: table of experiments for TiO2 layer optimization and collection of empiric responses, Table S4: inter-maps RSD of progressively increasing investigated areas.
