# Supplementary material for: Flexible and Transparent Substrates Based on Gold Nanoparticles and TiO2 for in Situ Bioanalysis by Surface-Enhanced Raman Spectroscopy
Source: Biosensors (Basel). 2019 Dec 17;9(4):145. doi: 10.3390/bios9040145 (PMC6955768; doi:10.3390/bios9040145)
Supplement: Supplementary file 1 [file biosensors-09-00145-s001.pdf]

*sensors*

MDPI

*Article*

# PDMS/TiO2/AuNPs Flexible Substrate for Food Contaminants Detection in Situ by Surface Enhanced Raman Scattering

**Luisa Mandrile [1], Andrea Mario Giovannozzi [1], Alessio Sacco [1], Gianmario Martra [1,2] and Andrea Mario Rossi [1,*]**

[1] Physical Chemistry and Nanotechnologies group, National Institute of Metrological Research, Strada delle Cacce 91, 10135 Turin, Italy; l.mandrile@inrim.it (L.M.); a.giovannozzi@inrim.it (A.M.G.); a.sacco@inrim.it (A.S.)

[2] Department of Chemistry and Interdepartmental Centre NIS, University of Turin, Via Giuria 7, 10125 Turin, Italy; gianmario.martra@unito.it

* Correspondence: a.rossi@inrim.it; Tel.: +390-113-919-342

Received: 15 November 2019; Accepted: 13 December 2019; Published: date

## 1. Characterization of synthetized AuNPs

The synthetized AuNPs were characterized for shape and dimensions using scanning electron microscopy, and the characteristic localized surface plasmon resonance frequency wavelength was measured by UV-vis absorption spectroscopy.

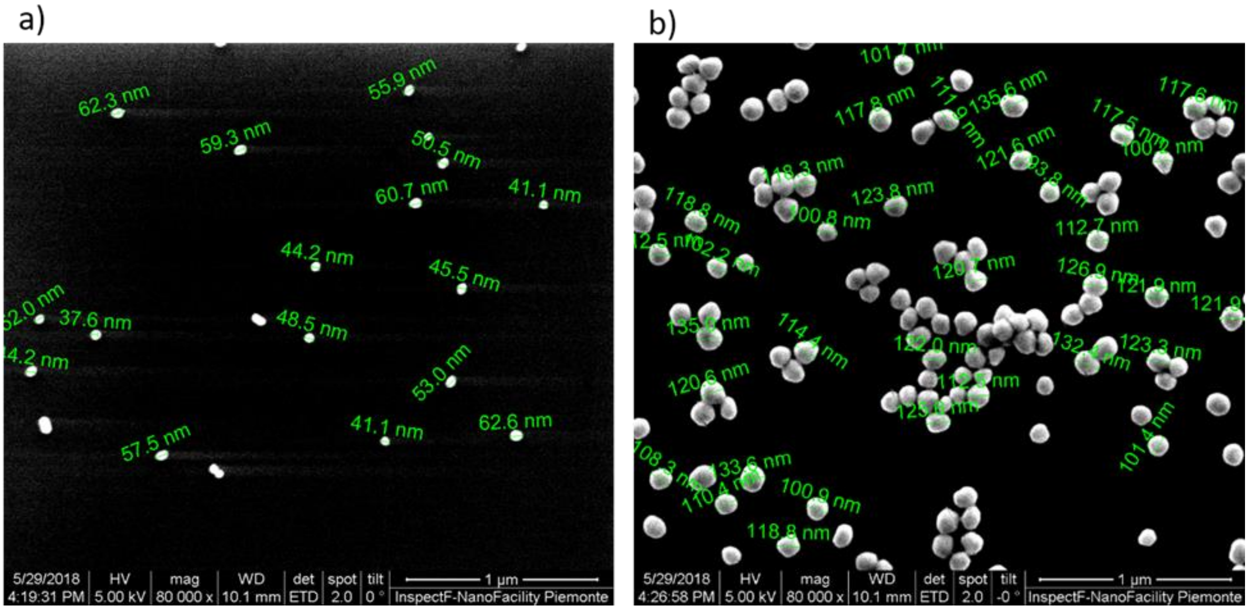


**Figure S1.** SEM images at magnification 80000× of AuNPs with nominal diameter of (**a**) 40 nm and (**b**) 120 nm deposited on a clean silicon wafer.

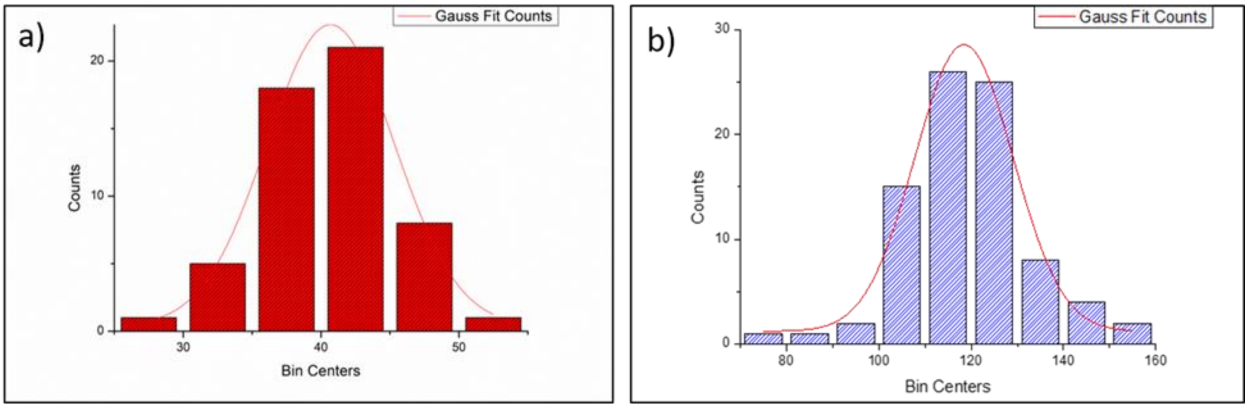


**Figure S2.** Size distribution of synthetized AuNPs with nominal diameter of (**a**) 40 nm and (**b**) 120 nm, calculated by the Gaussian fit of the diameters of 53 and 64 NPs observed by SEM, respectively.

**Table S1.** measured size of AuNPs by SEM.

| Average (nm) | St. Dev | FWHM | N |
|---|---|---|---|
| 40.7 | 4.7 | 10.9 | 53 |
| 116,42 | 10,8 | 11,6 | 64 |

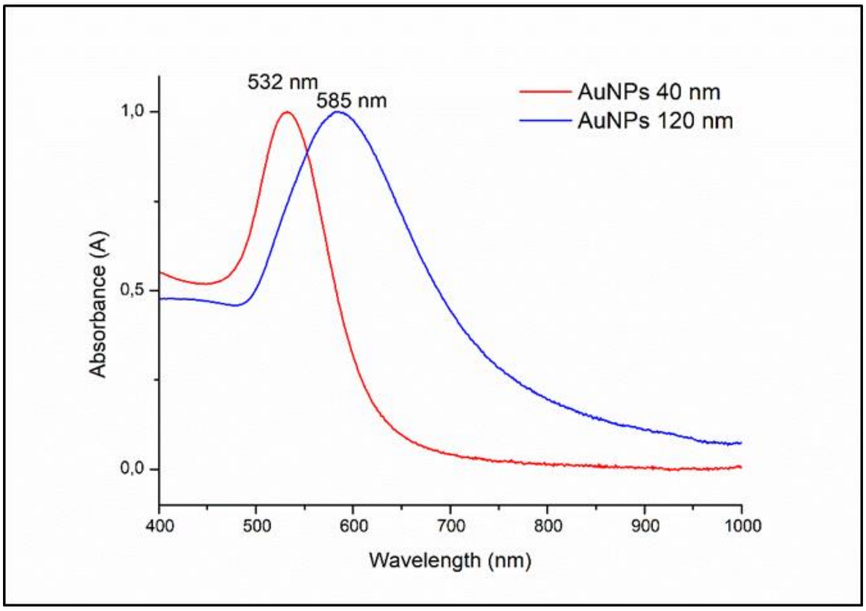


**Figure S3.** Visible absorption spectra of the synthetized AuNPs colloids in water.

## 2. Optimization of SERS tape preparation procedure

The Design of Experiments approach was used to determine the best conditions to obtain the desired features of the film. Three factors are considered in the optimization of the PDMS substrate preparation, i.e. the thickness (0.2 mm – 2 mm), the curing time (3 min – 15 min) and the microwave power (300 W – 700 W). The monitored responses were mechanical resistance and adhesion; these two features were evaluated on arbitrary performance scales. Marks ranging from 0 to 5 for mechanical resistance and from 0 to 10 for adhesion were assigned to each experiment. The exploited DoE method was D-optimal carried out using the Modde 7 Umetrics® software. 23 experiments were designed with different combinations of the varying parameters, three replicates for the central point were included in the experiment list to enhance the model stability. After the collection of experiment responses, multiple linear regression (MLR) coefficients were calculated and response curves were

elaborated to identify the optimal conditions for PDMS preparation. The experimental sheet with the corresponding results is reported in Table S2 in supplementary information.

**Table S2.** Table of experiments for PDMS optimization and collection of empiric responses.

| Exp No | Exp Name | Run Order | Thickness (mm) | Curing power (W) | Curing time (min) | Mechanical properties (Performance Mark 0-5) | Adhesion (Performance Mark 0-10) |
|---|---|---|---|---|---|---|---|
| **1** | N1 | 10 | 0.2 | 300 | 3 | 2 | 9 |
| **2** | N2 | 17 | 2 | 300 | 3 | 0 | 10 |
| **3** | N3 | 15 | 0.2 | 500 | 3 | 2 | 8 |
| **4** | N4 | 23 | 1.1 | 500 | 3 | 2 | 7 |
| **5** | N5 | 11 | 0.2 | 700 | 3 | 3 | 7 |
| **6** | N6 | 2 | 2 | 700 | 3 | 2 | 8 |
| **7** | N7 | 5 | 1.1 | 700 | 3 | 3 | 7 |
| **8** | N8 | 14 | 0.2 | 300 | 5 | 2 | 8 |
| **9** | N9 | 3 | 2 | 300 | 5 | 0 | 9 |
| **10** | N10 | 4 | 1.1 | 300 | 5 | 1 | 9 |
| **11** | N11 | 12 | 0.2 | 700 | 5 | 3 | 7 |
| **12** | N12 | 19 | 2 | 700 | 5 | 3 | 8 |
| **13** | N13 | 18 | 0.2 | 300 | 10 | 3 | 8 |
| **14** | N14 | 13 | 2 | 500 | 10 | 2 | 8 |
| **15** | N15 | 25 | 1.1 | 700 | 10 | 4 | 6 |
| **16** | N16 | 24 | 0.2 | 300 | 15 | 3 | 7 |
| **17** | N17 | 20 | 2 | 300 | 15 | 2 | 8 |
| **18** | N18 | 6 | 2 | 300 | 15 | 2 | 8 |
| **19** | N19 | 1 | 0.2 | 500 | 15 | 3 | 6 |
| **20** | N20 | 9 | 1.1 | 500 | 15 | 4 | 6 |
| **21** | N21 | 16 | 0.2 | 700 | 15 | 3 | 2 |
| **22** | N22 | 8 | 2 | 700 | 15 | 4 | 7 |
| **23** | N23 | 7 | 1.1 | 700 | 15 | 5 | 5 |
| **24** | N24 | 21 | 1.1 | 700 | 15 | 5 | 5 |
| **25** | N25 | 22 | 1.1 | 700 | 15 | 5 | 5 |

The MLR model for both responses show high correlation indexes, i.e. $R^2_{Mech\ prop}$= 0.95; $R^2_{Adhesion}$= 0.90. The real vs predicted values are reported in Figure S4. Good models are obtained as demonstrated by the symmetric random dispersion of the experimental values around the diagonal, which represents the perfect superimposition of real and calculated values. No evident outlier are identified.

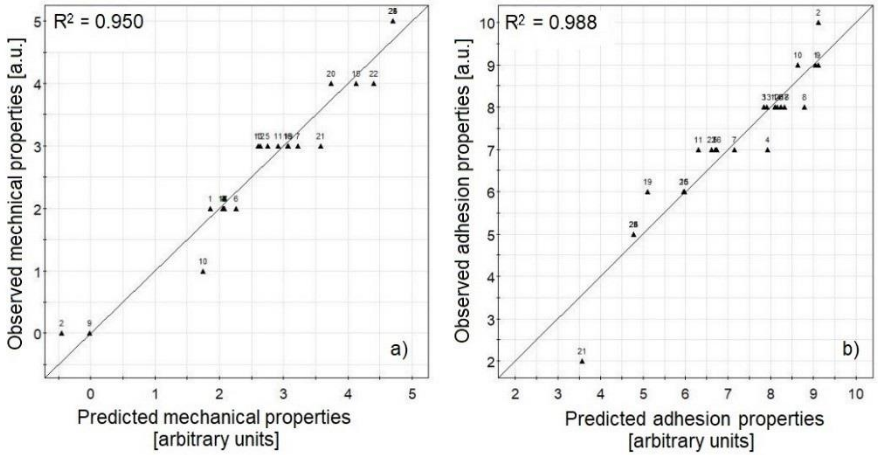


**Figure S4.** Observed versus predicted (**a**) mechanical properties and (**b**) adhesion performance marks calculated by MLR model.

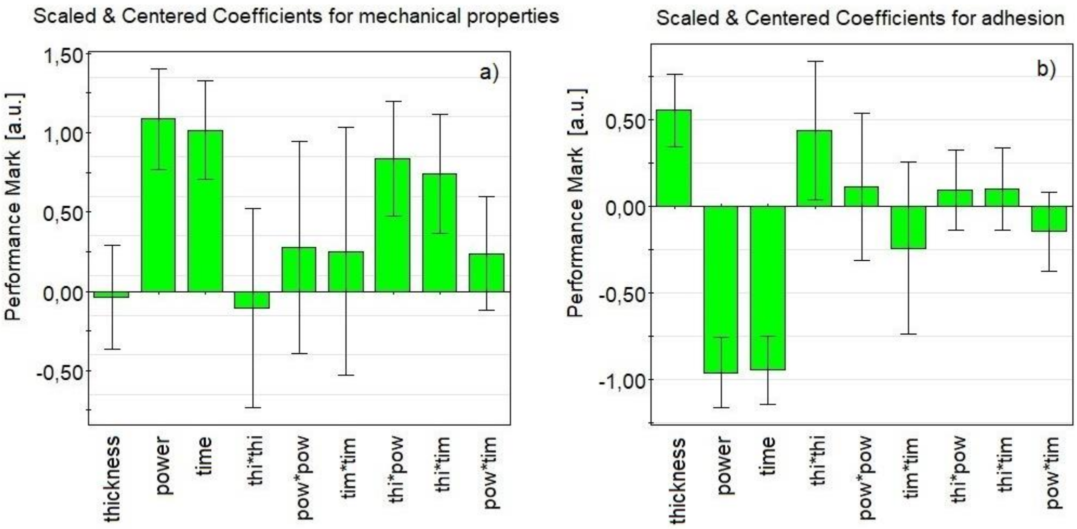


**Figure S5.** MLR model coefficients for (**a**) mechanical properties of PDMS and (**b**) adhesion.

The centered and scaled coefficients plot with confidence intervals are presented in Figure S5. The size of the coefficients represents the change in the response when a factor varies of one unit, while the other factors are kept at their averages. The coefficient is significant when the associated confidence bar does not cross the zero. For both responses the most relevant factors are power and time of curing, which provide opposite effects on mechanical resistance and stickiness of the obtained PDMS tape. The longer and more energetic was the curing, the more the tape was resistant. Conversely, very sticky PDMS was obtained with shorter and less energetic curing, as it was expected.

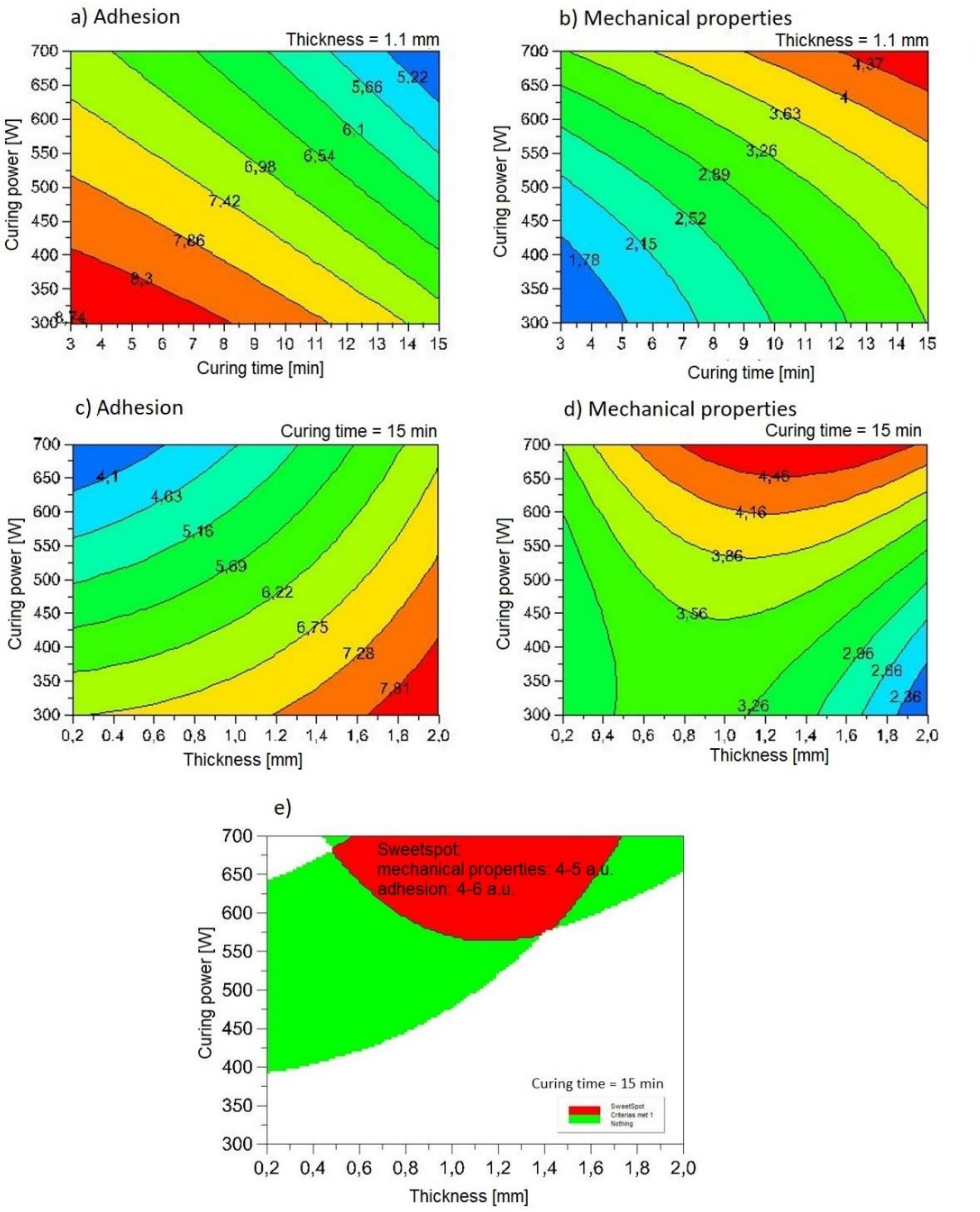


**Figure S6.** (**a, b, c, d**) response curves for adhesion and mechanical properties as function of thickness, curing power and curing time; (**e**) sweet spot plot for the identification of the best thickness and curing conditions of PDMS tape for SERS substrates application.

The DoE and subsequent modeling is very useful to define the best compromise parameters to satisfy contemporarily the two desired features by defining the region in the experimental space where performance indexes of both responses fulfill the expectations. The response curve plots for both responses are shown in Figure S6 a, b, c, b, and allow to identify the experimental conditions that provide desired results. The sweet spot plot is represented in Figure S3e, as the red-colored region in which all the responses show satisfactory levels. For this specific application, i.e. the fabrication of a flexible SERS tape to be laid down on testing surfaces, it should by sticky enough to adhere on the testing surface, but sufficiently consistent to allow easy handling with tweezers. In the arbitrary appreciation scale for adhesion from 0 (completely inconsistent and gluey) to 10 (perfectly solid and plastic), the desired optimum correspond to 5, whereas the mechanical properties should be maximized, and the desired value was 5. The superimposition of the response curves allows the identification of a sweet spot where both requirements are fulfilled, in particular the final protocol

for an optimal PDMS tape preparation consists in drop casting an amount of liquid PDMS in a petri dish adequate to obtain 1 mm thick PDMS layer, curing was carried out at maximum power (700 W) for 15 minutes. In this way reproducible sticky but consistent tapes were obtained.

A similar approach was used to determine the best $TiO_2$ deposition conditions. In this case the evaluated factors were $TiO_2$ paste concentration (5% – 20%) and dropped volume (5 μL – 15 μL). The operative conditions were kept constant in accordance with doctor blade published methodology (51, 52). Also in this case arbitrary appreciation scales are defined and performance indexes are attributed to different experimental results to evaluate the responses. Subsequently, a MRL model was calculated. The monitored responses were flexibility, i.e. absence of fractures on the dried films, adhesion, and diffusion, i.e. AuNPs homogenous distribution. The list of experiments is reported in Table S3 with the associated responses; replicates are included to test reproducibility and strengthen the model.

**Table S3.** Table of experiments for $TiO_2$ layer optimization and collection of empiric responses.

| Exp No | Exp Name | Run Order | Concentration (% w/w) | Volume (µl) | Flexibility (Performance Mark 0-10) | Adhesion (Performance Mark 0-10) | Diffusion (Performance Mark 0-10) |
|---|---|---|---|---|---|---|---|
| **1** | N1 | 13 | 5 | 5 | 10 | 10 | 1 |
| **2** | N2 | 12 | 5 | 5 | 10 | 10 | 1 |
| **3** | N3 | 22 | 10 | 5 | 9 | 9 | 10 |
| **4** | N4 | 3 | 15 | 5 | 7 | 8 | 10 |
| **5** | N5 | 16 | 20 | 5 | 3 | 6 | 10 |
| **6** | N6 | 11 | 20 | 5 | 3 | 6 | 10 |
| **7** | N7 | 4 | 5 | 8 | 10 | 10 | 3 |
| **8** | N8 | 5 | 10 | 8 | 9 | 9 | 9 |
| **9** | N9 | 18 | 15 | 8 | 7 | 7 | 10 |
| **10** | N10 | 14 | 20 | 8 | 3 | 5 | 10 |
| **11** | N11 | 20 | 5 | 12 | 9 | 9 | 3 |
| **12** | N12 | 19 | 10 | 12 | 9 | 9 | 9 |
| **13** | N13 | 2 | 15 | 12 | 4 | 4 | 10 |
| **14** | N14 | 10 | 20 | 12 | 3 | 4 | 10 |
| **15** | N15 | 6 | 5 | 15 | 10 | 9 | 2 |
| **16** | N16 | 8 | 5 | 15 | 10 | 9 | 2 |
| **17** | N17 | 9 | 10 | 15 | 8 | 7 | 10 |
| **18** | N18 | 17 | 15 | 15 | 4 | 3 | 10 |
| **19** | N19 | 7 | 20 | 15 | 2 | 2 | 10 |
| **20** | N20 | 23 | 20 | 15 | 2 | 2 | 10 |
| **21** | N21 | 15 | 20 | 15 | 2 | 2 | 10 |
| **22** | N22 | 1 | 20 | 15 | 2 | 2 | 10 |
| **23** | N23 | 21 | 20 | 15 | 2 | 2 | 10 |

The model obtained provided good correlation coefficients, $R^2_{Flexibility}$= 0.97; $R^2_{Adhesion}$= 0.96; $R^2_{Diffusion}$= 0.95. The experimental vs calculated values (Figure S7) provided a further proof of models goodness. The coefficients bar plots (Figure S8) attested that the considered factors, i.e. volume and concentration of titanium dioxide paste, play an opposite role as it was expected.

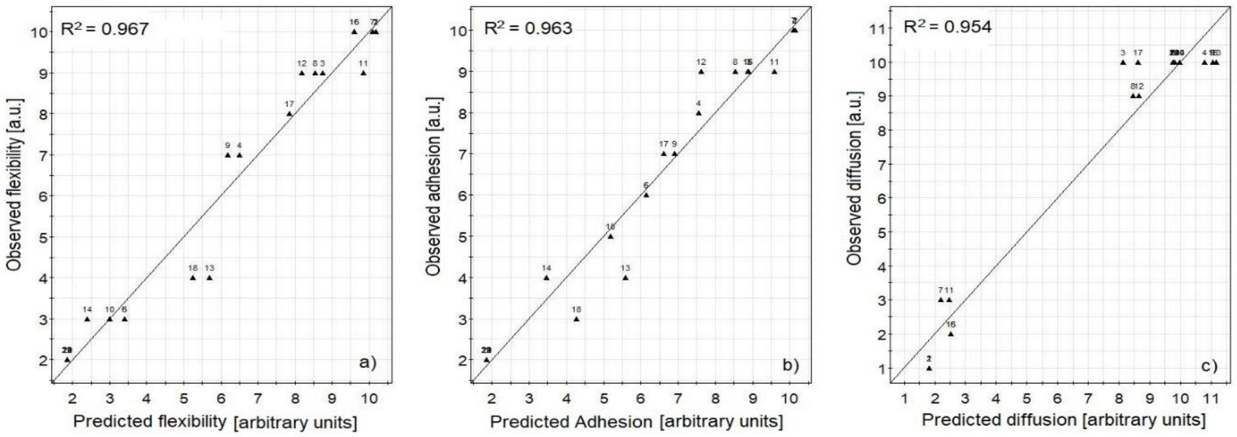


**Figure S7.** Observed vs predicted Performance marks for the three monitored responses (**a**) flexibility; (**b**) adhesion; (**c**) diffusion.

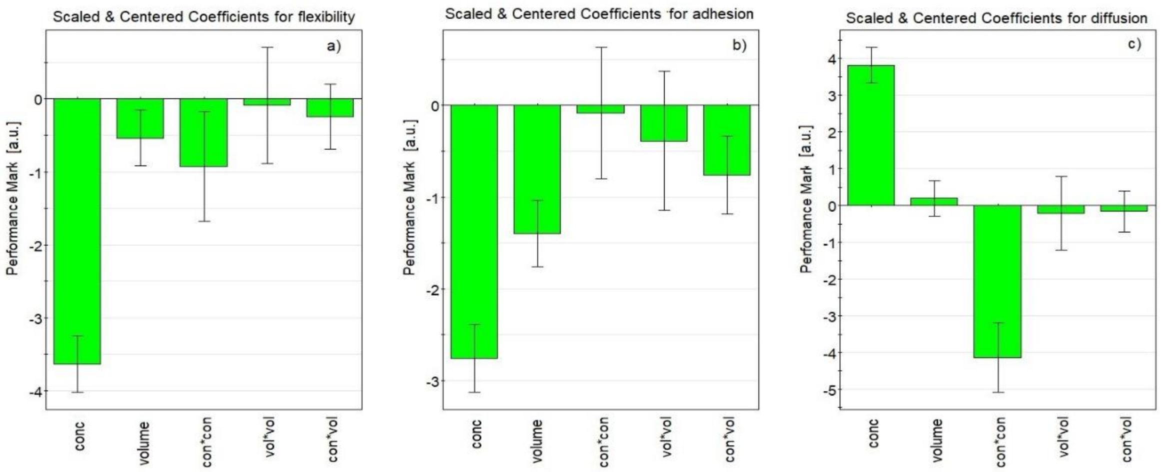


**Figure S8.** MLR model coefficients for (**a**) flexibility of $TiO_2$ layer; (**b**) adhesion of the tape after the deposition of the $TiO_2$ layer; (**c**) diffusion of AuNPs over the tape.

The experimental design was set to identify the so-called sweet spot, which is the set of experimental conditions that lead to optimal results.

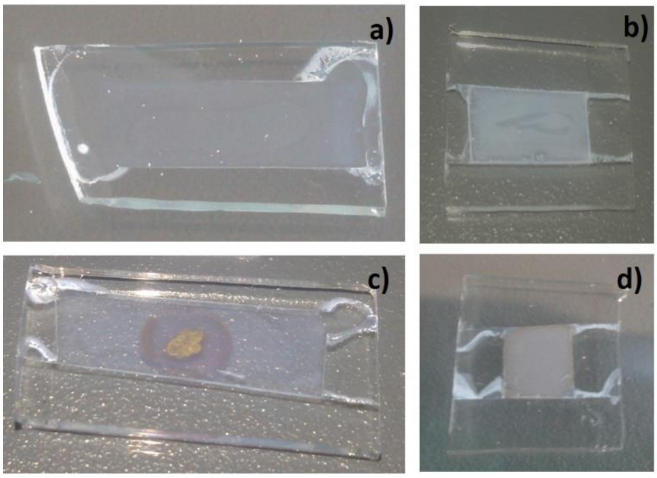


**Figure S9.** visual appearance of $TiO_2$ covered PDMS tape corresponding to undesired and desired experimental conditions. (**a, c**) thin and flexible $TiO_2$ layer obtained by depositing 8 µl 10% w/w paste before and after AuNPs deposition respectively; (**b, d**) thicker $TiO_2$ layer obtained by depositing 15 µl 20 % w/w paste, before and after AuNPs deposition respectively.

Thanks to the MLR model it was possible to define the proper concentration and amount of $TiO_2$ paste to be spread on the tape to meet the desired requirements, as shown in Figure S10, where the response curves and the sweet spot plot is reported. According to the experimental results, the selected preparation conditions for the titanium dioxide film deposition were a 10% w/w concentration, of which 8 μL are dropped and rapidly spread with a clean glass stick.

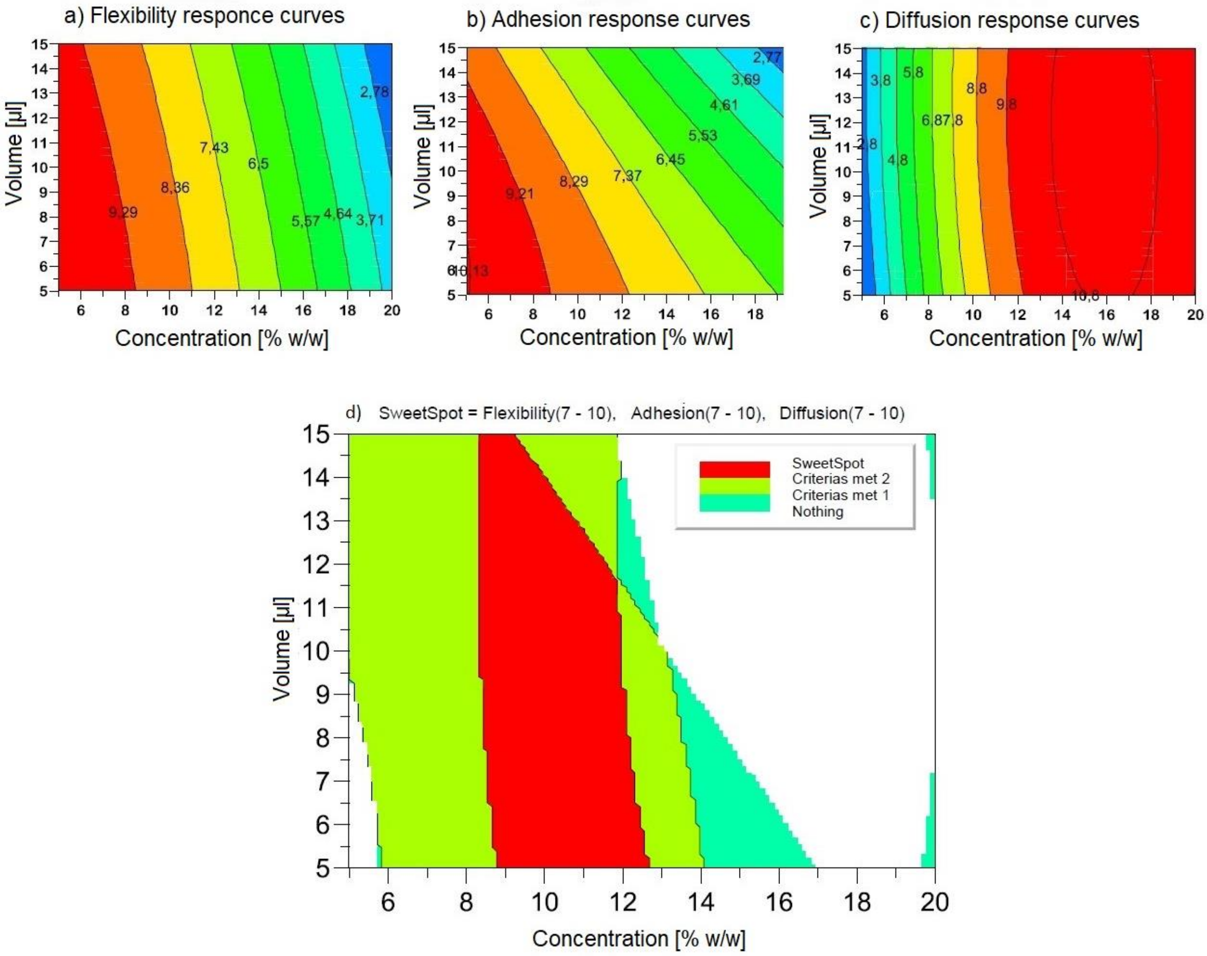


**Figure S10.** Response curves associate to the three monitored responses, (**a**) flexibility, (**b**) adhesion, (**c**) diffusion; (**d**) sweet spot plot for the optimization of the titanium dioxide layer.

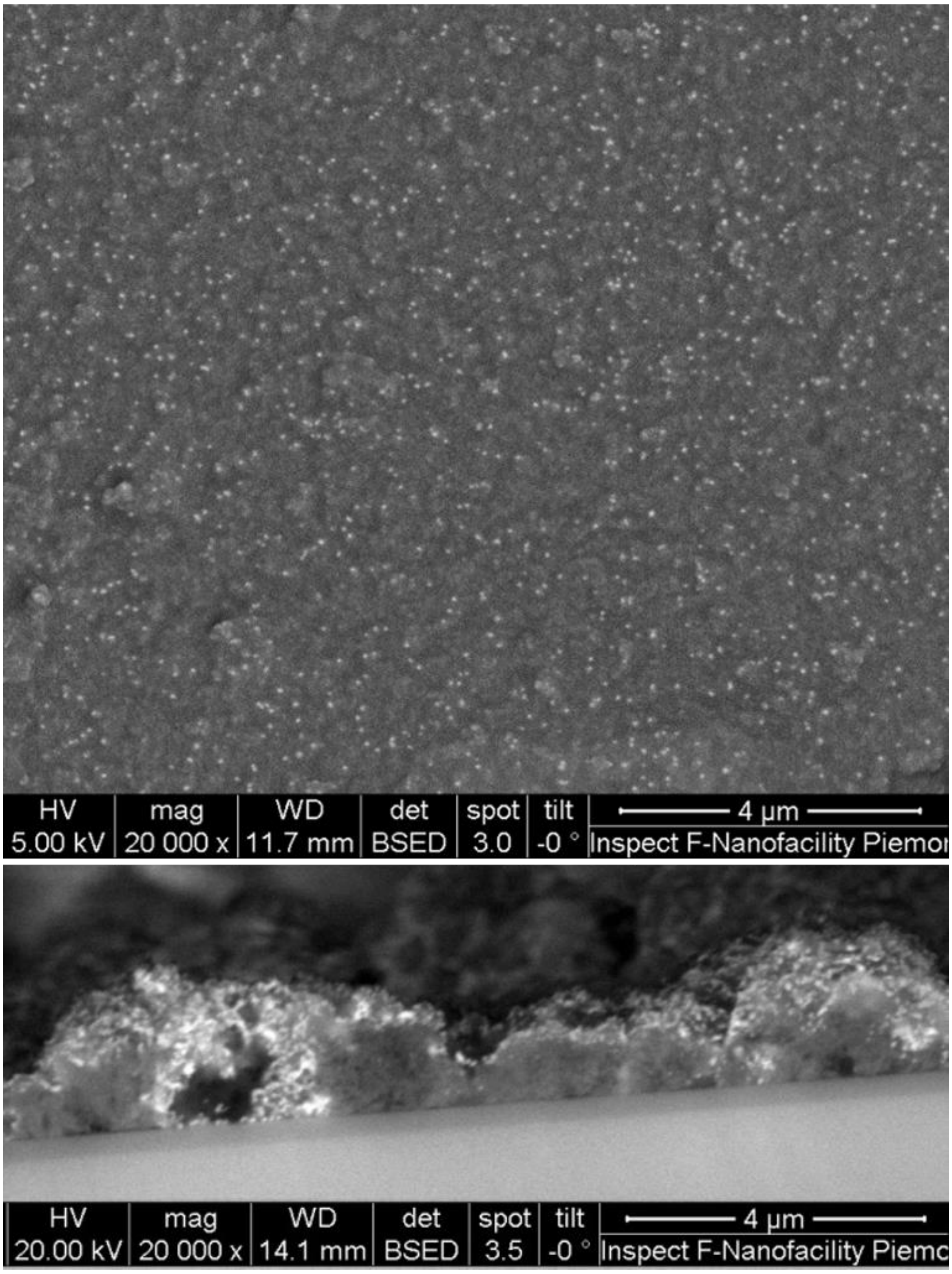


**Figure S11.** SEM images (back scattered electrons detector, 20000× magnification). AuNPs deposited on $TiO_2$/PDMS tape. (**a**) zenithal view; (**b**) cross sectional view.

7 mercapto-4-methylcumarin was selected as a Raman reporter because of its relatively large Raman cross section and its strong chemical affinity for gold due to the sulfhydryl functional group (R-SH) Raman spectrum of MMC in solid state is shown in Figure S9 with the characteristic peaks assignment. The band assignments were obtained by a combination of a computational procedure with vibrational information reported in literature (1, 2, 3). Geometry optimization of model MMC structures and consequent calculations of vibrational spectra were carried out with DFT method using Gaussian 03 program (Gaussian 03, Revision B.05, References cited in http://www.gaussian.com). Full geometry optimizations were carried out without symmetry constraints. The computations were performed with the Lee, Yang and Parr correlation functional (LYP) (4) combined with the Becke's non-local three-parameter hybrid exchange functional. Vibrational information coming from the computational procedure were compared with the

experimental Raman spectrum of MMC and the main bands in the spectrum were assigned using the Handbook of Infrared and Raman Spectroscopy (5).

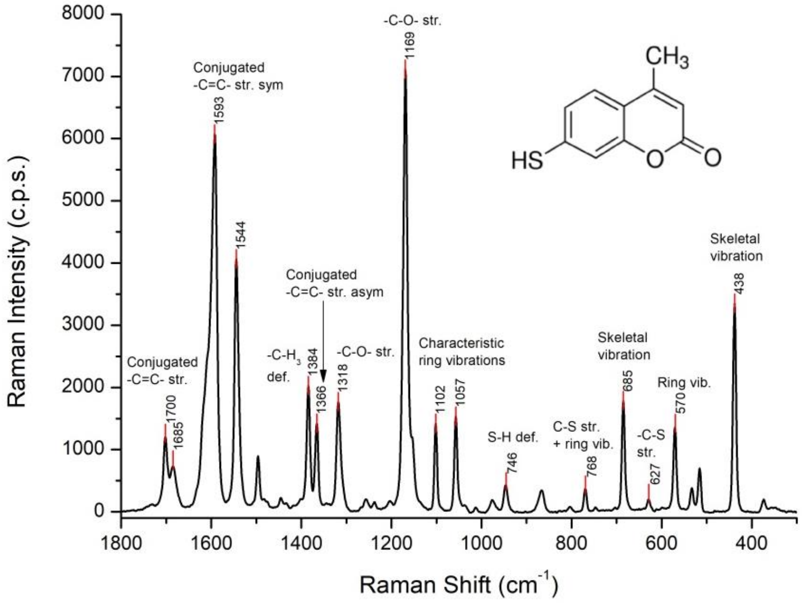


**Figure S12.** Raman spectrum of 7-mercapto-4-methylcoumarin (MMC) in solid state with the characteristic peaks assignments.

## 3. Substrate homogeneity and response repeatability

The uniformity of the SERS response over an active substrate is a crucial aspect in view of a real application; in order to evaluate the variability of the signal across the substrate, the relative standard deviation (RSD) was evaluated. The RSD is defined as the percent ratio of the standard deviation to the mean, and it is widely adopted in the SERS community to assess the spatial homogeneity of a substrate. Lower RSD values show remarkable homogeneity (6) and very low values have been recently reported (7). However, a standard protocol to evaluate spatial homogeneity of SERS substrates is still missing. Sometimes the RSD is calculated by considering the average of tens to thousands punctual spectra (8, 9), whereas it may be obtained from multiple scanning areas on the substrate (10). Moreover, the considered area onto which the analysis was carried out is not clearly declared, leading to non-comparable results (11, 12). For the purpose of defining the SERS substrates homogeneity for analytical and bioanalytical applications over large area, the analysis should be extended to greater portions of the substrate and the analyzed area should be stated unequivocally. First, the intra-map homogeneity, which is described by the RSD calculated on all the spectra composing the Raman map, was calculated. In other words, it represents the variability from pixel to pixel within one single map. As long as the scanned area is enlarged, the response variability increases, leading to a higher RSD value. On the other hand, repeatability of the measurements can be obtained by increasing the spot size of analysis: in this way, local differences are averaged. In punctual confocal Raman, the spot size is a constant which depends on the section of the focalized laser on the investigated surface, which is 2.7 µm for a 780 nm laser and a 0.25 NA 10× objective. However, a spot size enlargement can be practically obtained collecting spectra on a wider area and averaging all of them. In this way, the point-to-point intensity differences can be overcome. The resulting mean spectrum is representative for the whole mapped area. The intra and inter maps RSDs of the three replicates are reported in Table 1.

The minimum area that guarantees adequate repeatability was investigated. The RSD of repeated measurements of equal areas on the same SERS substrate, i.e. the inter-maps RSD, is

considered to evaluate this. For this scope, the calculation of the RSDs was performed by considering the SERS spectra acquired over three analogue samples. Three model surfaces covered by a MMC monolayer are covered with a SERS tape and measured using SERS mapping. For each sample the SERS map is repeated considering progressively increasing areas as reported in Table S4 and Figure S13. It can be noticed that as long as the scanned area is larger, the variation of the results obtained for three repeated measurements gets lower and lower. An area of 0.25 $mm^2$ is demonstrated to be optimal to guarantee good measurement repeatability since 10% RSD is obtained, however adequate measurement repeatability is reached from 0.1 $mm^2$ on. Moreover, it can be noticed that for scanned areas higher than 0.1 $mm^2$ the accuracy of the results is also improved, to narrow investigated areas can lead to inaccurate intensity determinations since the investigation may be not representative enough.

**Table S4.** Inter-maps RSD of progressively increasing investigated areas.

| Map size | Area | I(mean) rep1 | I(mean) rep2 | I(mean) rep3 | I(mean) Inter-map | st.dev | Inter map RSD % |
|---|---|---|---|---|---|---|---|
| 5x5 px | 0.016 $mm^2$ | 198 | 400 | 363 | 320 | 88 | 27 |
| 8x8 px | 0.040 $mm^2$ | 365 | 413 | 264 | 348 | 62 | 18 |
| 10x10 px | 0.063 $mm^2$ | 377 | 415 | 286 | 359 | 54 | 15 |
| 13x13 px | 0.106 $mm^2$ | 299 | 367 | 384 | 350 | 37 | 11 |
| 15x15 px | 0.141 $mm^2$ | 311 | 409 | 350 | 357 | 40 | 11 |
| 18x18 px | 0.20 $mm^2$ | 348 | 396 | 310 | 351 | 35 | 10 |
| 20x20 px | 0.25 $mm^2$ | 319 | 399 | 337 | 352 | 34 | 10 |

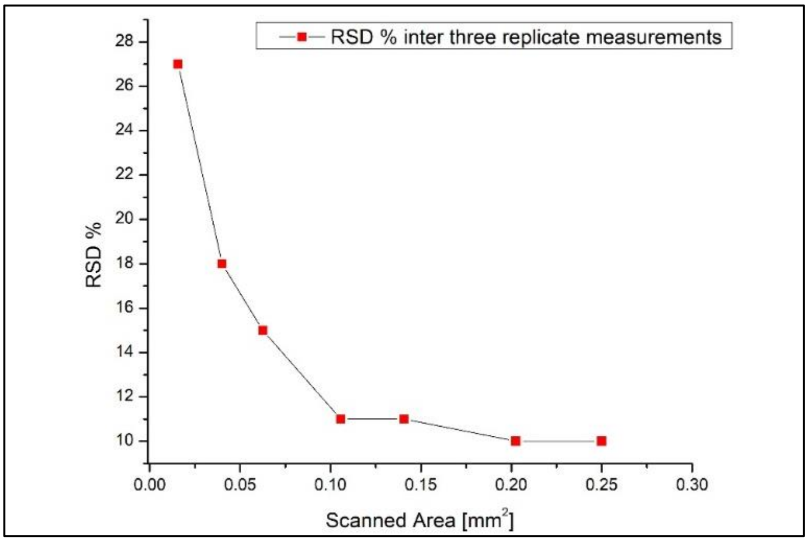


**Figure S13.** Trend of the RSD% calculated inter 3 measurements as a function of the size of the mapped area.

## 4. Enhancement factor

To establish the values for $I_{Raman}$ a 0.01 M ethanol solution of MMC was poured into a well. With a 20× LWD objective and using an excitation wavelength of 780 nm and a power of 8 mW (20 s exposure time), 5.2 counts/s ($I_{Raman}$) were measured at the 1595 $cm^{-1}$ peak. Using the interaction volume of 68 $\mu m^3$, the number of molecules responsible for the Raman signal ($N_{Raman}$) was estimated to be $4 \times 10^9$.

To establish the values for $I_{SERS}$, a SERS tape coated with AuNPs was incubated in 4 ml of MMC $10^{-4}$ M for 4 hours, then abundantly rinsed and dried. 3 Raman maps were collected on 6 different samples using identical conditions as for $I_{Ref}$ a signal intensity of 2200 ± 400 counts/s was measured for the 1595 $cm^{-1}$ peak. The number of molecules inside the laser spot was estimated by assuming a monolayer of MMC all over the AuNPs surface. Knowing the AuNPs concentration ($2.7 \times 10^{-10}$ mol/l)

and the diameter of one spheroidal NP (116 ± 11 nm), hypothesizing a uniform distribution of AuNPs on the surface (as proved by SEM imaging and SERS homogeneity tests) and a laser spot-size with diameter 1.9 µm an effective active area available for SERS was (2.7 ± 0.5 µm$^2$). This gives an estimate of $N_{SERS}$ = 4.2 × 10$^6$ ± 0.3 × 10$^6$). Hence the calculated enhancement factor comes to 3.4 x 10$^5$ ± 0.4 × 10$^5$.

$$\boldsymbol{EF} = \frac{\boldsymbol{Isers} \times \boldsymbol{Nraman}}{\boldsymbol{Iraman} \times \boldsymbol{Nsers}} \tag{1}$$

$$\boldsymbol{Isers} = measured\ on\ a\ SERS\ tape\ incubated\ substrate$$

(with a monolayer of MMC molecules on the surface of each NP)

$$\boldsymbol{Iraman} = measured\ in\ a\ wll\ containing\ 0.01\ M\ MMC\ solution\ (5{,}167\ cps)$$

$$\boldsymbol{Nraman} = NA \times Volume\ Ellipsoide(cm^3) \times [MMC]\ (mol/cm^3)$$

$$[MMC] = \frac{mass(g)}{MM}(g/mol)/V(ml)$$

$$Volume\ Ellipsoide = 4/3\pi \times r^2 \times h$$

$$\boldsymbol{Nsers} = \frac{Asers}{Amolecule\ (assuming\ a\ coverage\ factor\ of\ the\ surface = 1)}$$

$$Amolecule = (Molecule\ Radius \times 2)^2$$

$$Molecule\ raduis = \sqrt[3]{(3 \times Vmolecule/4\pi)}$$

$$Vmolecule = Vmolar/NA$$

$$Vmolar = \frac{MM}{densityof\ MMC\ (g/cm3)}$$

$$\boldsymbol{Asers} = SupAreaNPs(cm^2)(4\pi r^2) \times number\ of\ NPs\ in\ the\ spot$$

$$number\ of\ NPs\ in\ the\ spot = n\ NPs\ deposited\ on\ the\ tape \times Spot\ Size\ Area\ (cm^2)$$

The analytical enhancement factor was also evaluated in the "use configuration". The EF was obtained by applying the previously described equation.

$$EF = I_{SERS}C_{Raman}/I_{Raman}C_{SERS}$$

The intensity of the MMC peak at 1595 cm$^{-1}$ was used from the spectra and the control test flat spectrum in Figure 3 to get the $I_{SERS}$ and $I_{Raman}$, respectively. Since no Raman signal of the MMC was collected without the SERS tape, due to the low sensitivity of the traditional Raman in the detection of a monolayer of organic molecules, the noise of the control spectrum, intended as the standard deviation of the random oscillation of the baseline in correspondence of the non-present peak, i.e. spectral region 1750 cm$^{-1}$ – 1550 cm$^{-1}$, was considered at the $I_{Raman}$ term. Assuming that number of MMC molecules was the same for SERS and Raman measurements the terms $C_{SERS}$ and $C_{Raman}$ are removed. The average EF calculated out of three determinations is 2.43 × 10$^2$ with a RSD of 9.7%.

## References


1. Erdogdu, Y., Saglam, S. Infrared, Raman and NMR spectra, conformational stability and vibrational assignment of 7, 8-Dihydroxy-4-Methylcoumarin. Spectrochim. Acta, Part A, **2014**, *132*, 871–878.
2. Arivazhagana, M., Sambathkumarb, K., Jeyavijayanc, S. Density functional theory study of FTIR and FT-Raman spectra of 7-acetoxy-4-methyl coumarin. Indian J. Pure Appl. Phys. **2010**, *48*, 716–722.
3. Socrates, G. *Infrared and Raman characteristic group frequencies: tables and charts*. John Wiley & Sons: Hoboken, NJ, USA, 2004.
4. Lee, C. Yang, W and Parr R.G. Development of the Colle-Salvetti correlation-energy formula into a functional of the electron density. Phys. Rev. B **1988**, *37*, https://doi.org/10.1103/PhysRevB.37.785.
5. Lewis, I. R., Edwards, H. *Handbook of Raman spectroscopy: from the research laboratory to the process line*. CRC Press: Boca Raton, FL, USA, 2001.

6. Caridad, J. M., Winters, S., McCloskey, D., Duesberg, G. S., Donegan, J. F., Krstić, V. Hot-Volumes as Uniform and Reproducible SERS-Detection Enhancers in Weakly-Coupled Metallic Nanohelices. Sci. Rep. **2017**, *7*, 45548.
7. Lin, D., Wu, Z., Li, S., Zhao, W., Ma, C., Wang, J., Jiang, Z., Zhong, Z., Zheng, Y. and Yang, X. Large-Area Au-Nanoparticle-Functionalized Si Nanorod Arrays for Spatially Uniform Surface-Enhanced Raman Spectroscopy. ACS nano, **2017**, *11*, 1478–1487
8. Chen, H. Y., Lin, M. H., Wang, C. Y., Chang, Y. M., Gwo, S. Large-scale hot spot engineering for quantitative SERS at the single-molecule scale. JACS **2015**, *137*, 13698–13705.
9. Huang, J.A., Zhao, Y.Q., Zhang, X.J., He, L.F., Wong, T.L., Chui, Y.S., Zhang, W.J. and Lee, S.T. Ordered Ag/Si nanowires array: wide-range surface-enhanced Raman spectroscopy for reproducible biomolecule detection. Nano Lett. **2013**, *13*, 5039–5045.
10. Peksa, V., Lebrušková, P., Šípová, H., Štěpánek, J., Bok, J., Homola, J., & Procházka, M. Testing gold nanostructures fabricated by hole-mask colloidal lithography as potential substrates for SERS sensors: sensitivity, signal variability, and the aspect of adsorbate deposition. PCCP **2016**, *18*, 19613–19620.
11. Shuyuan, C., Jun, L., Wei, W. Preparation of metal nanoparticles-based conductive inks and their applications in printed electronics. Process. Chem. **2015**, *27*, 1509–1522.
12. Jamil, A. K., Izake, E. L., Sivanesan, A., Agoston, R., Ayoko, G. A. A homogeneous surface-enhanced Raman scattering platform for ultra-trace detection of trinitrotoluene in the environment. Anal. Methods **2015**, *7*, 3863–3868.
13. Green, M., and Liu, F.M. SERS substrates fabricated by island lithography: the silver/pyridine system. J. Phys. Chem. B **2003**, *107*, 13015–13021


© 2020 by the authors. Submitted for possible open access publication under the terms and conditions of the Creative Commons Attribution (CC BY) license (http://creativecommons.org/licenses/by/4.0/).
